# Proceedings of the Sixth Annual Meeting of the National Confederation of State Medical Examining and Licensing Boards, Held at Atlanta, Ga., May 4, 18961From the Bulletin of the American Academy of Medicine, June, 1896.

**Published:** 1896-07

**Authors:** Benj. M. Griffith

**Affiliations:** Secretary


### Society Proceedings.

### PROCEEDINGS OF THE SIXTH ANNUAL MEETING OF THE NATIONAL CONFEDERATION OF STATE MEDICAL EXAMINING AND LICENSING BOARDS, HELD AT ATLANTA, GA., MAY 4, 1896.[1]

THE president, Dr. WILLIAM WARREN POTTER, of Buffalo, called the meeting to order at 10 o'clock A. M., in the assembly room of the Hotel Aragon, and introduced Dr. J. C. OLMSTEAD, member of the Georgia State Board of Medical Examiners, who welcomed the confederation in the following words :

*Mr. President :* On behalf of the medical profession of Atlanta and the Board of Medical Examiners of this state, it gives me pleasure to welcome you to this, our "Cracker City of the Red Hills," and to the glorious old state of Georgia. The presence in our midst of your distinguished body is indeed an honor that we deeply appreciate. Representing as you do a legislative force emanating from our own profession and of comparatively recent date, which has become most potent in its influence for good upon medical education and qualification, your conventions, proceedings and wise councils must ever command the interest, sympathy and high respect of all who have at heart the true welfare, honor and elevation of the medical profession.

The influence for good and the consecration of the public weal attained through many obstacles, despite the political demagoguery and prejudice inspired generally by ignorant or selfish men, in the institution of State Medical Examining Boards, has already been so clearly demonstrated that we have good hope that the day is not far distant when every state of our glorious union will have its independent, unprejudiced examining board, which shall stand as an impregnable bulwark in defense of the ignorant and weak against the selfishness and ambition of those known by the name of physician only.

And as the tendency of modern science, like the political status of our country, is toward unity and a completed whole, so in ours, the most liberal of professions, the harmony, purpose and unity of design point toward the same goal. We should aim to accomplish the inauguration of a system of state medical examinations that shall know no North or South, no East or West, but which shall recognise only the perfected harmony of a united whole. This I understand to be your chief aim and object—namely, by the confederation and unity of our honored profession, in its efforts to benefit mankind and advance the interests of its

1. From the *Bulletin of the American Academy of Medicine*, June, 1896.

own noble purpose, shall by its devotion to the welfare of its fellow-men when truly followed entitle it to Ben Adhem's motto, "leads all the rest."

Trusting your sojourn among us may be conducive to the pleasure and advancement of your association, again, gentlemen, permit me to welcome you to our hearts and homes.

The President invited Dr. James Mackintosh Hays, of Greensboro, N. C., Vice-President of the Confederation, to respond. Dr. Hays spoke as follows:

*Mr. President:* Through the partiality of our distinguished presiding officer, the pleasant duty has been assigned me of touching the button, so to speak, which is to set the machinery of this meeting in motion. I feel that I should be recreant to my trust, however, were I not first to thank the distinguished gentleman who has so cordially welcomed us to this beautiful city and extended to each of us its entire freedom. While, therefore, we are all grateful to him for this kind welcome, it really seems, in a certain sense, almost a work of supererogation to extend the hospitality of this city to any particular sect; for, within the last twelve months have we not all seen this "Gateway of the Sunny South" thrown wide open to every nation and kindred, tongue and tribe? And has not the whole world here enjoyed a hospitality in which the land of "Dixie" is found to excel?

Atlanta is not the pride of Georgia alone. Each of our states has its capital: Virginia is proud of her Richmond; the old North state points with a warm interest to her little capital nestling among the ancient and grand old oaks; but Atlanta is the pride of the whole South, and we glory with the Empire state in her magnificent schools, her medical colleges, her argentiferous *Constitution* and her auriferous *Journal*, her wonderful railroad facilities and her enormous mercantile establishments. Let me also congratulate Dr. Olmstead and his colleagues in their skilful fostering of obstetrical cases which enables Atlanta to double her population with clockwork regularity every ten years; so that while thanking Dr. Olmstead on behalf of our organisation for his charming words of welcome, I feel that I must be allowed also to join with him in extending the hospitality of this beautiful city to those of you who are not fortunate enough to have a home beneath our Southern skies.

And, Mr. President, permit me to say in this connection that you are now among a people who, while loving and revering the "conquered banner" as a sacred memory, are as loyal as any in this broad land to "Old Glory." Let but the call for volunteers under the old flag ever be made and the nations of the earth will be astonished at the magnificent response from our Southern boys. There is no state this side of Mason and Dixon's line that would not be glad to accept the commission singly and alone, at this very moment, to free poor bleeding Cuba from the mercenary grasp of that heartless monarchy whose name is synonymous with greed and gore.

Our meeting here, at this time, Mr. President, is peculiarly auspicious. We see representatives of the North and South, the East and West assembled in this room with a single purpose—namely, to elevate the standard of education in the noblest of all professions.

There is much to be accomplished. Our peculiar work is in almost virgin soil. I am proud of the fact that I represent the state which was the pioneer in establishing a separate examining board. The laws of the various states that have laws at all differ widely. The question of reciprocity should engage our most serious attention. It is not at all improbable that this very organisation will solve the problem of universal state laws—a consummation devoutly to be wished.

One of the most serious questions before us, too, is that of preliminary education. New York has set a splendid pace in this regard, and other states are very properly making an attempt to regulate that matter. It is the most serious problem that confronts us in North Carolina.

I had thought to touch on several of the more important subjects which should come before us, but by reference to the program I notice that the ground is wellnigh covered by able thinkers, and as time is precious I will now give way that the regular order may proceed.

At this point the confederation went into joint session with the American Academy of Medicine and the American Association of Medical Colleges, for the purpose of listening to a discussion on Medical education, arranged by the American Academy of Medicine, Dr. Henry M. Hurd, president, in the chair.

AFTERNOON SESSION—2.30 O'CLOCK.

The vice-president, Dr. James Mackintosh Hays, in the chair.

The president, Dr. William Warren Potter, then delivered his annual address, choosing for his subject Relations of medical examining boards to the state, to the schools and to each other. (See age 938.)

Mr. James Russell Parsons, Jr., of Albany, Director of Examinations University of the State of New York, upon invitation, then delivered an address on Preliminary education, professional training and practice in New York.

At the conclusion of Mr. Parsons's address, Dr. Charles A. L. Reed, of Ohio, moved that the thanks of the confederation be tendered to Mr. Parsons for his able, comprehensive and instructive address. The motion was unanimously carried.

Dr. JOSEPH M. MATHEWS, of Louisville, expressed his high appreciation of Mr. Parsons's address and tendered his personal thanks to Mr. Parsons for coming such a long distance to attend this meeting and to present so superbly the work done by the great State of New York in advancing the standard of medical education. He wished to announce, however, that the smaller, yet great State of Kentucky, had also done a great work, though in a different manner. During a recent journey to Chicago and New York he was amazed to see upon almost every street corner advertisements of charlatans and quacks. And yet Illinois and New York had done almost more than any other states to improve educational methods in regard to medicine. He could, however, in contrast point with pride to Kentucky, where 1,150 quacks and charlatans had been driven across her borders and now Kentucky was free from the humiliation of their presence.

This announcement was greeted with great applause.

Dr. MATHEWS then read a paper entitled Limitations of the standard of modern educational requirements as determined by state medical examining boards.

Dr. CHARLES McINTIRE, of Easton, Pa., next read a paper entitled Some obstacles to an inter-state recognition of a state license to practise medicine, with suggestions for their removal.

The VICE-PRESIDENT: We have listened with great pleasure and profit, I am sure, to the four carefully prepared papers that have been read this afternoon, all bearing more or less upon the same subject. The whole question is now open for consideration and I request Dr. Vander Veer to open the discussion thereon.

Dr. A. VANDER VEER, of Albany: Mr. Chairman, the four papers cover the ground so completely that it is almost impossible for anyone to take up each paper separately for discussion in a way that can add materially to what has already been said. Permit me to say, however, that I was greatly interested in each paper.

About fifteen years ago an effort was made, in which I took an active part, to have a law passed to bring about a more uniform system of teaching in the State of New York. After struggling four years with the legislature we finally succeeded in securing the passage of a law providing for the registration of physicians. It was well received by the profession, which encouraged us to go further. Looking to Illinois as our guide and citing the action of the Illinois state board of health as an illustration, we sought to impress the members of our legislature with the necessity of passing a law that would relieve the embarrassment presented by the fact that medical students would come from adjoining states and after attending two courses of lectures, of from ten to sixteen weeks each, would graduate and begin practice alongside of our own physicians, who had received much longer training. We first attempted to obtain a mixed board of state examiners, but the legislature said to us: "We have passed laws recognising three distinct medical societies in this state. We will encourage the passing of a law giving you a board representing each state medical society." After carefully considering this proposition the three several state societies, representing regular, homeopathic and eclectic medicine, were authorised to nominate each a state board of medical examiners. The appointing power was placed in the hands of the regents of the University of the State of New York. This law took effect in 1891, and we have been operating under it ever since. The results have been carefully and ably set forth by Mr. Parsons here today. In our last legislature no less than twenty-three bills relating to medical affairs were introduced, some of which were of a vicious nature, and one of which was a stealthy attempt to modify our present law relating to the preliminary requirements for medical students. Out of this group but one bill passed, however, which was the bill referred to by Dr. Potter and Mr. Parsons in their addresses, which establishes a four years' course after January 1, 1898.

I believe the members of our legislature are now alert and thoroughly alive to the necessity of supporting our medical practice act, hence in future it will not be easy for designing persons to secure improper amendments or additions. It will not be difficult, it seems to me, for other states to secure similar legislation, provided the medical profession sets itself at work on solid lines, manifesting an earnest desire to obtain good and wholesome laws and impressing the several legislatures with the importance thereof.

Our president gave us some excellent points in his address regarding the preliminary acquirements, medical collegiate training and final examination of students. I believe Dr. Potter presented very clearly and concisely the views of the profession in the State of New York on the subjects dealt with, and I commend them to the careful consideration of the members of this body.

Dr. William S. Foster, of Pittsburg: I have been most pleasantly and profitably entertained in listening to the different addresses and essays bearing on the several points pertaining to state medical examination for license that have been presented here today. In Pennsylvania we have adopted the New York standard of preliminary requirements, and we have a medical council, which, to all intents and purposes, exercises the function of the New York Board of Regents in the supervision of the issuance of licenses to practise medicine. I regret, however, that we have no protection in Pennsylvania against the quack unless he does some criminal act, and thus far it has been impossible to remedy this defect through adequate legislation.

Dr. Charles A. L. Reed, of Cincinnati: It is a matter of very great regret on my part that I was unable to hear all the papers in their entirety, but I may be permitted to remark, judging from expressions I hear on all hands, that we are all moving on convergent lines toward the accomplishment of a definite purpose —namely, that of uniform state requirements for the legalising of the practice of medicine in this country. Having had but little opportunity of judging, I do not feel competent to discuss the details of this movement, but I beg you all to consider that we are not dealing with theories, but with conditions; that these conditions vary in different particulars in different states, and that each state and community presents its own peculiar problem. Finally, though I am fully committed to the principle of uniformity of methods and standards, I yet must urge that we move slowly and judiciously toward the accomplishment of that end, to which purpose I pledge my best efforts.

The papers were further discussed by Drs. J. E. Chancellor and R. W. Martin, of Virginia, and Mr. James Russell Parsons, Jr., of Albany.

The committee on constitution and revision of by-laws presented its report, which was unanimously adopted. (See *Bulletin, American Academy of Medicine*, December, 1895, page 350, also the Journal, December, 1895, p. 407, for full report of this committee.)

The report of the secretary and treasurer was then read and made part of the proceedings of the meeting.

The chair having been duly authorised, appointed a committee of nomination, consisting of Dr. Perry H. Millard, of Minnesota, Dr. Joseph M. Mathews, of Kentucky, and Dr. William S. Foster, of Pennsylvania.

This committee subsequently reported the following-named as officers for the ensuing year, who were unanimously elected:

president, William Warren Potter, M. D., Buffalo, N. Y.; vice-presidents, Charles A. L. Reed, M. D., Cincinnati, O., J. N. McCormick, M. D., Bowling Green, Ky.; secretary and treasurer, A. Walter Suiter, M. D., Herkimer, N. Y.; executive council, Perry H. Millard, M. D., St. Paul, Minn., Joseph M. Mathews, M. D., Louisville, Ky., William S. Foster, M. D., Pittsburg, Pa., Hugh M. Taylor, M. D., Richmond, Va., Jas. Mackintosh Hays, M. D., Greensboro, N. C.

Dr. Perry H. Millard moved that a committee of five on minimum standard of requirements, to report next year, be authorised.

The president appointed the following committee on the minimum standard of requirements: Dr. Perry H. Millard, Minn., chairman; Drs. N. R. Coleman, O.; B. M. Griffith, Ill.; J. M. Hays, N. C., and Gardiner T. Swarts, R. I.

The following-named were elected to membership: Honorary, James Russell Parsons, Jr., Albany, N. Y.; William Osler, M. D., Baltimore, Md. Active, Charles McIntire, M. D., Easton, Pa.; John B. Roberts, M. D., J. C. Wilson, M. D., Frank Woodbury, M. D., H. A. Hare, M. D., Philadelphia, Pa.; A. Vander Veer, M. D., Albany, N. Y.; J. D. Spencer, M. D., Watertown, N. Y.; A. W. Suiter, M. D., Herkimer, N. Y.; H. M. Paine, M. D., West Newton, Mass.; Wm. E. B. Davis, M. D., Birmingham, Ala.; Geo. H. Rohé, M. D., Henry M. Hurd, M. D., Baltimore, Md.; J. A. Larrabee, M. D., C. W. Kelly, M. D., J. M. Bodine, M. D., D. S. Reynolds, M. D., L. S. McMurtry, M. D., J. A. Ouchterlony, M. D., Louisville, Ky.; Henry R. Hopkins, M. D., Buffalo. N. Y.; Hunter McGuire, M. D., Geo. Ben Johnston, M. D., Richmond, Va.; N. S. Davis, M. D., E. Fletcher Ingals, M. D., John B. Hamilton, M. D., Chicago, Ill.; Daniel Lewis, M. D., Albert L. Gihon, M. D., L. Duncan Bulkley, M. D., New York City, N. Y.; Brigadier-General Geo. M. Sternberg, Surgeon-General U. S. Army, Walter Wyman, M. D., Supervising Surgeon-General U. S. M. H. S., Washington, D. C.; W. F. Westmoreland, M. D., Atlanta, Ga.; John O. Roe, M. D., Rochester, N. Y.; J. D. Griffith, M. D., Kansas City, Mo.; E. S. Pettyjohn, Alma, Mich.; Geo. W. Stoner, M. D., U. S. M. H. S.

The next annual meeting was appointed at Philadelphia, to convene at 10 o'clock, A. M., Monday May 31, 1897.

The thanks of the Confederation were then tendered: First, to Dr. W. F. Westmoreland, chairman committee of arrangements, for many courtesies shown to the Confederation and its several members during the meeting; second, to Mr. C. F. Dodge, manager of the Hotel Aragon, for the many comforts and conveniences provided the members, and for the free use of the assembly room, in which the meeting was held.

The president expressed himself as deeply grateful to the gentlemen who had come great distances to read papers and discuss the subjects presented at this meeting. This organisation, he said, though yet in its infancy, gave promise of increasing usefulness, and he hoped in bidding the members good-bye to meet them all in Philadelphia next year.

The meeting was then adjourned *sine die*.

BENJ. M. GRIFFITH, M. D., *Secretary*.

